# Mais Estudos devem ser Realizados sobre o Volume Vascular Pulmonar Estimado por Software Automatizado em Embolia Pulmonar Aguda

**DOI:** 10.36660/abc.20200959

**Published:** 2020-11-01

**Affiliations:** 1 Complexo Hospitalar de Niterói Radiologia Cardiovascular NiteróiRJ Brasil Complexo Hospitalar de Niterói - Radiologia Cardiovascular, Niterói. RJ - Brasil; 2 Universidade Federal Fluminense Hospital Universitário Antônio Pedro Departamento de Radiologia NiteróiRJ Brasil Universidade Federal Fluminense Hospital Universitário Antônio Pedro - Departamento de Radiologia, Niterói, RJ – Brasil

**Keywords:** Embolia Pulmonar Aguda/complicações, Prognóstico, Medicina de Precisão, Tomografia Computadorizada/métodos, Algoritmos, Software, Inteligência Artificial/tendências

A embolia pulmonar aguda (EPA) tem apresentação clínica heterogênea e o desenvolvimento de ferramentas clínicas para melhor estratificação do prognóstico dos pacientes é bastante desejável. Ser capaz de discernir qual paciente tem maior probabilidade de ter complicações ou até mesmo morrer é fundamental na era da medicina de precisão. Isso pode auxiliar na escolha de um tratamento mais intensivo e individualizado.^1^

A angiotomografia pulmonar (ATCP) já é o método mais utilizado em emergências para o diagnóstico e estratificação de pacientes com EPA. A grande variabilidade das apresentações clínicas, seja em pacientes com câncer ou em diferentes condições trombogênicas, torna alguns dados quantitativos mais atrativos do que apenas a análise qualitativa do exame.^2^^–^^5^

Assim, o trabalho de Soriano et al.^6^ que desenvolve um algoritmo de análise de dados totalmente automatizado, capaz de extrapolar parâmetros que diferenciam os indivíduos com maior probabilidade de morrer em um curto período de tempo, é de grande valor.^6^

O principal resultado é que o volume vascular pulmonar ajustado (VVPa) diminuiu significativamente no grupo de não-sobreviventes em comparação ao grupo de sobreviventes (21 ± 6 cm^3^/L vs. 30 ± 7 cm^3^/L, p = 0,001).^6^ E o melhor ponto de corte do VVPa para determinar a mortalidade em um mês foi de 23 cm^3^/L.^6^

Nesta etapa, eu gostaria de levantar pontos específicos que são fundamentais para uma boa leitura e compreensão do direcionamento que este trabalho nos fornece.

O tamanho da amostra provavelmente não foi suficiente para termos o poder de concluir que esse método pode ser extrapolado para outras populações, mas serve para antecipar uma possível tendência e estimular novos estudos. Como discutido pelo grupo, a maioria dos estudos já publicados recrutou um número muito maior de participantes, como Coutance et al.,^7^ com 383 pacientes com EPA, Moroni et al.^8^ com 225 ATCP com EPA não grave e Kamamaru et al.,^9^ com 1.698 ATCP de pacientes com EPA.

Há um claro viés de seleção porque o código de embolia pulmonar (I26) não foi utilizado como critério de seleção no registro do banco de dados do hospital utilizando a Classificação Estatística Internacional de Doenças (CID-10). Neste estudo, temos apenas os códigos referentes à embolia pulmonar com *cor pulmonale* aguda (I26.0) e embolia pulmonar sem *cor pulmonale* aguda (I26.9).

Além disso, dos 84 estudos sobre ATCP recuperados, apenas 61 (73%) puderam ser analisados de forma automatizada pelo *software* Yacta. Geralmente, pacientes com casos mais graves, maior dispneia e instabilidade hemodinâmica serão aqueles que terão conjuntos de dados com mais artefatos, dificultando o uso do algoritmo do Yacta.

Outro ponto no qual ainda tenho dúvida é a utilização desse método para diferentes tipos de tomógrafos computadorizados. Portanto, estou satisfeito em ver o desenvolvimento alcançado, mas ainda vejo muitas questões a serem respondidas em um estudo prospectivo e multicêntrico com tomógrafos de múltiplos detectores.

Por fim, espero que o uso de inteligência artificial ajustada a um banco de dados possa melhorar a eficiência do software, reduzindo os casos atualmente excluídos. Também acredito que este banco de dados pode ser usado para categorizar os achados. Que venham mais estudos!!!
