# Supplementary material for: Mais Estudos devem ser Realizados sobre o Volume Vascular Pulmonar Estimado por Software Automatizado em Embolia Pulmonar Aguda
Source: Arq Bras Cardiol. 2020 Nov 1;115(5):819–20. [Article in Portuguese] doi: 10.36660/abc.20200959 (PMC8452191; doi:10.36660/abc.20200959)
Supplement: Translation

# Translation: Ventricular Repolarization as a Tool to Monitor Electrical Activity of the Heart

**Affiliations:** 1 NiteróiRJ Brazil Complexo Hospitalar de Niterói - Radiologia Cardiovascular, Niterói. RJ - Brazil; 2 NiteróiRJ Brazil Universidade Federal Fluminense Hospital Universitário Antônio Pedro - Departamento de Radiologia, Niterói, RJ – Brazil

**Keywords:** Acute, Pulmonary Embolism/complications, Prognosis, Precision Medicine, Compute Tomography/methods, Algorithms, Software, Artificial Intelligence/trends

Acute pulmonary embolism has a heterogeneous clinical presentation and the development of clinical tools for better stratification of the patients prognosis is extremely desired. Being able to discern which patient is most likely to have complications or even death is critical in the age of precision medicine. This may assist in choosing a more intensive and individualized treatment.^1^

Computed tomography pulmonary angiography (CTPA) is already the most used method in emergencies for the diagnosis and stratification of patients with APE. The great variability of clinical presentations, whether in patients with cancer or different thrombogenic conditions, makes some quantitative data more attractive than just the qualitative analysis of the exam.^2^^–^^5^

Thus, the work of Soriano et al.^6^ which develops a fully automated data analysis algorithm capable of extrapolating parameters that differentiate individuals most likely to die in a short period of time, is of great value.^6^

The main result is the adjusted pulmonary vascular volume (aPVV) was significantly decreased in the non-survivors group in comparison to the survivor's group (21±6 cm^3^/L vs. 30±7 cm^3^/L, p=0.001).^6^ And the best cutoff point of the aPVV to determine the one-month mortality was 23 cm^3^/L.^6^

At this stage, I would like to raise specific points that are fundamental for a good reading and understanding of the direction that this work provides us.

The sample size was probably not enough to have power to conclude that this method can be extrapolated to other populations, but it serves to advance a possible trend and encourage new studies. As discussed by the group, most of the studies already published recruited a much larger number of participants such as Coutance et al.^7^ with 383 patients with APE, Moroni at al.^8^ with 225 CTPA with non severe APE and Kamamaru et al.^9^ with 1698 CTPA from APE patients.

There is a clear selection bias because the pulmonary embolism (I26) code was not used as selection criteria in the hospital database record using the International Statistical Classification of Diseases (ICD-10). In this study, we have only the codes referring to pulmonary embolism with acute cor pulmonale (I26.0) and pulmonary embolism without acute cor pulmonale (I26.9).

Also, from 84 retrieved CTPA studies, just 61 (73%) were possible to be automated analyzed by the Yacta software. Generally, patients with more severe cases, greater dyspnea and hemodynamic instability will be those who will have datasets with more artifacts, making it difficult for the Yacta's algorithm.

Another point that I still doubt is the use of this method for different types of computed tomography scanners. Therefore, I am pleased to see the development achieved, but I still see many questions to be answered in a prospective and multicentric trial, with multivendor CT scanners.

Finally, I hope that the use of artificial intelligence adjusting to a database should be able to improve the software efficiency reducing the cases that are currently excluded. I also believe that this database may be used to categorize the findings. Let more studies come!!!
